# The prevalence and incidence of community-acquired pressure injury: A protocol for systematic review and meta-analysis

**DOI:** 10.1097/MD.0000000000022348

**Published:** 2020-11-25

**Authors:** Geng Chen, Lv Lin, Yang Yan-Lin, Chung Yuet-Foon Loretta, Lin Han

**Affiliations:** a Evidence-based Nursing Center, School of Nursing, Lanzhou University; b Wound Ostomy Care Center; c Department of Nursing, Gansu Provincial Hospital, Lanzhou, China.

**Keywords:** community-acquired pressure injury, incidence, meta-analysis, prevalence

## Abstract

**Background:**

Pressure injury (PI) is a serious problem in health care settings globally. It leads to tremendous burden both individuals and healthcare systems. Since 2008, hospital-acquired pressure injuries have been a major focus of nursing quality improvement programs within hospitals and are considered never events. However, insufficiency attention has been paid to community-acquired pressure injuries (CAPI) or pressure ulcers that occur at home or in nursing homes. The prevalence or incidence of community-acquired pressure injury has been reported but never been synthesized in a meta-analysis manner. To fill the gaps in the evidence matrix, the aims of this study are to estimate the prevalence of CAPI in the general population and to pool the overall incidence of CAPI in the general population.

**Methods:**

PubMed, Web of Science, EMBASE, CINHAL, the Cochrane Library, Chongqing VIP, and China National Knowledge Infrastructure were electronically searched to identify eligible studies updated to May 2020 to collect studies on the prevalence or incidence of community-acquired pressure injuries. Two reviewers independently will screen the literature, extracted data, and assess the risk of bias of included studies using the Strengthening the Reporting of Observational Studies in Epidemiology (STROBE) guideline. Meta-analyses of pooled weighted estimates will be calculated using random effect models with 95% CIs reported due to high heterogeneity.

**Results:**

Of the 5242 studies initially identified, of the 22 studies (total 479,761 participants) 17 reporting prevalence of community-acquired pressure injury and 5 reporting incidence were included. Other results of this study will be published in a peer-reviewed journal.

**Conclusion:**

This study will summarize the pooled estimate prevalence and incidence of community-acquired pressure injuries and the pooled estimate of frequencies of different anatomic sites.

**Ethics and dissemination:**

Ethics approval and patient consent are not required, because this study is a meta-analysis based on published studies.

**INPLASY registration number:**

INPLASY202080044

## 1 Introduction

Pressure injury (PI) is a serious problem in health care settings globally and it affects the health of more than 7 million people worldwide.^[1]^ Pressure injury is a localized injury to the skin and/or underlying tissue, usually over a bony prominence or related to a medical or other device and it is the result of intense and/or prolonged pressure or pressure in combination with shear.^[2]^ It leads to tremendous burden both individuals and healthcare systems.^[3]^ For instance, PI causes considerable patient suffering from pain,^[4]^ affects the patient's quality of life emotionally, physically, and socially,^[5,6]^ and even increases patient's risk of death.^[7]^ In addition, PI leads to an economic burden on health care systems such as it carries an estimated annual cost of $11 billion (US $).^[1,8]^

Since 2008, hospital-acquired pressure injuries (HAPI) have been a major focus of nursing quality improvement programs within hospitals and are considered never events.^[9]^ In the last few decades, pressure injury studies have mainly focused on HAPI. Systematic review studies show a wide range of PU prevalence rates among hospitalized patients: 3.1% to 30.0% in the United States, 1% to 54% in Europe, 6% in Australia, and 2.7% to 16.8% in Asia.^[10,11]^ Various preventive measures and treatments have been implemented in hospitals to reduce the prevalence of PI around the world.^[12]^ However, insufficiency attention has been paid to community-acquired pressure injuries (CAPI) or pressure ulcers that occur at home or in nursing homes. In many cases, PI has already been developed prior to hospital admission.^[13–15]^ A study executed in New England (n = 1022) addresses that 70.6% of the patients who already had PI before hospital admission were living at home before entering acute care hospital, and only 21.4% were receiving home care services prior to admission.^[14]^ Other studies have shown that the prevalence of community-acquired PI ranged from 3.3% to 11.1%.^[13,15–17]^ The prevalence or incidence of community-acquired pressure injury has been reported but never been synthesized in a meta-analysis manner.

To fill the gaps in the evidence matrix, we conducted a systematic review to retrieve epidemiological studies that reported the prevalence or incidence of CAPI. The aims of this study are to estimate the prevalence of CAPI in the general population and to pool the overall incidence of CAPI in the general population.

## 2 Methods

We have registered the protocol on the International Platform of Registered Systematic Review and Meta-analysis Protocols (INPLASY), and the registration number was INPLASY202080044. This systematic review will be conducted and reported in accordance with the Preferred Reporting Items for Systematic reviews and Meta-Analyses (PRISMA) guidelines.^[18]^

### 2.1 Eligibility criteria

#### 2.1.1 Types of patients

General population will be included. There will also be no restrictions based on other conditions, such as age, gender.

#### 2.1.2 Types of studies

We will consider observational studies such as cohort, case control, and cross-sectional study.

#### 2.1.3 Types of PI

PI diagnostic criteria according to NPUAP and there is no limit to the stage of PIs. PI should occur in non-hospital settings such as communities, nursing homes, and so on.

#### 2.1.4 Types of outcome measures

The primary outcomes are prevalence or incidence of CAPI in the general population.

### 2.2 Search methods and the identification of studies

#### 2.2.1 Electronic searches

**Figure 1:**
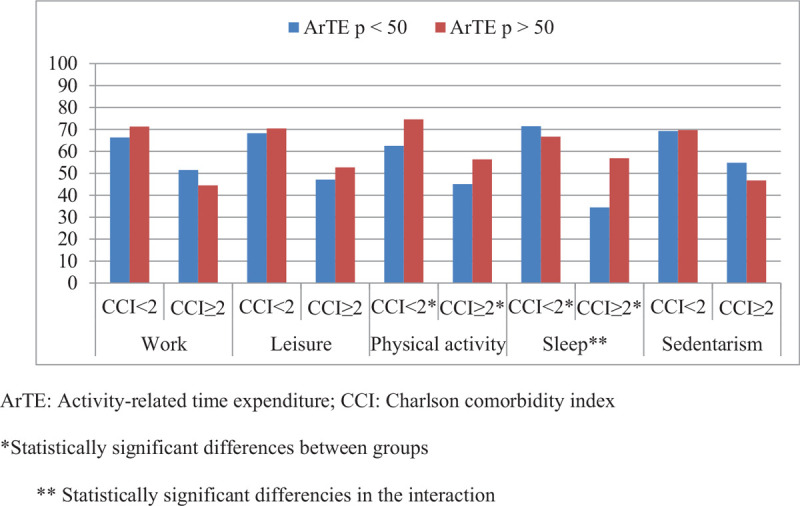
Flow chart of searching and screening studies.

Seven electronic databases (PubMed, Web of Science, EMBASE, CINHAL, the Cochrane Library, Chongqing VIP, and China National Knowledge Infrastructure) were systematically searched by the first author (CG) for all studies published from the earliest record to May 2, 2020 reporting the prevalence or incidence of community-acquired pressure injury among general population. The search terms were combinations of epidemiology (prevalence, incidence, or epidemiology), PI (pressure injury or pressure ulcer), and community-acquired (community, home, nursing home, residence home, long-term care center, or rehabilitation center) in forms of free words or controlled vocabulary (i.e., medical subject headings). There were no time or language limitations. The specific search strategies for PubMed bibliographic database are listed in Table 1. And the flow chart of searching and screening studies is shown at Figure 1

**Table 1:** Search strategy used in the PubMed database.

Search number	Search term
#1	(((((((((pressure ulcer^∗^) OR pressure injury) OR pressure sore^∗^) OR pressure damage) OR decubitus ulcer)) OR “Pressure Ulcer”[Mesh]))
#2	((“Community-Acquired Infections”[Mesh]) OR (((((((Community-acquired) OR community) OR nursing home^∗^) OR home) OR long-term care) OR residence home^∗^) OR rehabilitation center^∗^)))
#3	((((“Prevalence”[Mesh] OR “epidemiology” [Subheading]) OR “Incidence”[Mesh])) OR (((((prevalence) OR incidence) OR frequency) OR occurrence) OR rate))
#4	#1 AND #2 AND #3

#### 2.2.2 Searching other resources

In addition, we were also searched for dissertations and gray literature to identify systematic reviewers or clinical trials related to prevalence or incidence of CAPI. Besides, related journals and conference processes will be manually searched.

### 2.3 Data collection and analysis

#### 2.3.1 Selection of studies and data extraction

Initial search records will be imported into ENDNOTE X9 literature management software, then the titles and abstracts of records will be screened to identify potential trials according to eligibility criteria. Next, full-text versions of all potentially relevant trials will be obtained and reviewed to ensure eligibility.

A standard data extraction form will be created using Microsoft Excel 2013 to collect data of relevant information, including study characteristics (author[s], year of publication, study setting, year of investigation, study design, sampling method, assessment and diagnosis of PI) and data on prevalence (sample size and number of cases) or incidence (sample at risk and number of new cases). In addition, to locate potentially relevant studies that had been omitted the researchers screened the reference lists of the identified articles. Authors will be contacted if further information was needed.

Study selection and data extraction will be performed by 1 reviewer (GC), and will be checked by other reviewers (YYL, LL). Any conflicts will be resolved by discussion.

#### 2.3.2 Assessment of risk of bias

The quality of included studies will be assessed by using the Strengthening the Reporting of Observational Studies in Epidemiology (STROBE) guideline.^[19]^ The assessment included 5 modules, namely, sample population, sample size, participation rate, outcome assessment, and analytical methods. Each module was graded as with high risk and unclear (score 0), moderate risk (score 1), or low risk (score 2) (see Table 2). The overall bias risk of each study will be represented by the total score of the 5 modules.

**Table 2:** Quality score scale for assessing the risk of bias.

Bias type	Low risk (score = 2)	Moderate risk (score = 1)	High risk (score = 0)
Selection (sample population)	Sample from general population, not a select group; consecutive unselected population; rationale for case and control selection explained	Sample selected from large population but selection criteria not defined; sample selection ambiguous but may be representative; rationale for cases and controls not explained; eligibility criteria not explained; analysis to adjust for sampling strategy bias.	Highly select population making it difficult to generalize finding; sample selection ambiguous and sample unlikely to be representative.
Selection (sample size)	Sample size calculation performed and adequate	Sample size calculation performed and reasons for not meeting sample size given; sample size calculation not performed but all eligible persons studied.	Sample size estimation unclear or only subsample studied.
Selection (participation rate)	High response rate (>85%).	Moderate response rate (70–85%).	Low response rate (<70%); response rate not reported.
Performance bias (outcome assessment)	Diagnosis using consistent criteria and direct examination.	Assessment from administrative database or register; assessment from hospital record or interviewer	Assessment from nonvalidated data or generic estimate from the overall population
Performance bias (analytical methods to control for bias)	Analysis appropriate for the type of sample (subgroup analysis/regression etc.)	Analysis does not account for common adjustment	Data confusing

Two reviewers (GC and YYL) will independently assess the risk of bias for each study as low, moderate, or high using the STROBE. All disagreements in the review stage and data extraction process will be resolved by consensus through discussion.

#### 2.3.3 Statistical analysis

Before pooling prevalence estimates of CAPI, we will first assess the heterogeneity among studies using the Cochran's Q statistic and *I*^2^ index (the proportion of total variability due to true between-study heterogeneity beyond chance).^[20–22]^ A random-effects meta-analysis will be employed a priority throughout this study because of inherent variations between study characteristics (e.g., investigated sample, study design, and study location). All statistical analyses will be conducted with STATA version 12.0. A *P* value of less than .05 indicated statistical significance.

The subgroup analysis will be performed to investigate the possible sources of heterogeneity according to the stage of PIs, different regions, and different setting. The influence of a single study will be checked by a leave-one-out sensitivity analysis.^[23,24]^ We also will examine publication bias by visual inspection of funnel plots, Egger regression test for funnel plot asymmetry, and Begg rank correlation test.^[25–27]^

The prevalence of pressure injury will be calculated as the number of patients with pressure injury divided by the total number of inpatients on the ward. The incidence of pressure injury will be calculated as the number of patients with pressure ulcers that newly developed over the past month divided by the total number of inpatients.

## 3 Discussion

To the best of our knowledge, this is the first meta-analysis protocol for integrating the incidence and prevalence of community-acquired pressure ulcers in various countries and regions around the world. This study will integrate and compare the prevalence or incidence of pressure injuries in different regions and places, hoping that health care providers will pay more attention to the community-acquired pressure injury, and take timely targeted treatment measures. This protocol is designed in adherence to guidelines for meta-analysis protocols and will be conducted and reported strictly according to the PRISMA extension statement for network meta-analysis.

## Acknowledgments

The authors are grateful for the helpful reviewer comments on this paper.

## Author contributions

Chen Geng, Lin Lv and Lin Han tested the feasibility of the study.

Chen Geng, Lin Lv wrote the manuscript; all authors approved the final version of the manuscript.

Chen Geng, Lin Lv, Loretta Yuet-Foon Chung, Yanlin Yang and Lin Han provided methodological advice, polished, and revised the manuscript.

Chen Geng, Lin Lv: plan and design the research.

**Conceptualization:** Chen Geng.

**Data curation:** Chen Geng, Lin Lv.

**Formal analysis:** Chen Geng.

**Investigation:** Lin Han.

**Methodology:** Chen Geng, Lin Lv, Yanlin Yang and Lin Han.

**Resources:** Chen Geng, Lin Lv, Loretta Yuet-Foon Chung.

**Software:** Chen Geng, Yanlin Yang.

**Supervision:** Lin Lv, Lin Han.

**Validation:** Loretta Yuet-Foon Chung and Yanlin Yang

**Writing – original draft:** Chen Geng, Lin Lv.

**Writing – review & editing:** Lin Lv, Loretta Yuet-Foon Chung and Lin Han.
